# Just a spoonful of metformin helps the medicine go down

**DOI:** 10.1172/JCI179144

**Published:** 2024-03-15

**Authors:** Theophilos Tzaridis, Robert J. Wechsler-Reya

**Affiliations:** 1 Cancer Genome and Epigenetics Program, NCI-Designated Cancer Center, Sanford Burnham, Prebys Medical Discovery Institute, La Jolla, California, USA.; 2 Department of Neurology and Herbert Irving Comprehensive Cancer Center, Columbia University Medical Center, New York, New York, USA.

## Abstract

Diffuse intrinsic pontine glioma (DIPG) is a devastating brain tumor with a need for novel therapies. So far, monotherapies have failed to prolong survival for these patients, and combinatorial strategies have often shown severe, dose-limiting toxicities. In this issue of the *JCI*, Duchatel, Jackson, and colleagues address this challenge by introducing a drug combination that mitigates side effects and overcomes resistance. After identifying the PI3K/mTOR pathway as a therapeutic vulnerability, they treated DIPG-bearing mice with paxalisib and saw responses but also observed hyperglycemia as a severe side effect. Combining paxalisib with metformin mitigated this toxicity, but also upregulated protein kinase C (PKC) signaling. To tackle this mechanism of resistance, the authors added the PKC inhibitor enzastaurin to their drug combination and showed that this triple therapy led to improved survival. This approach paves the way for improved outcomes for patients with DIPG and other brain tumors.

## Balancing safety and efficacy in treatment of DIPG

The Swiss physician and philosopher Paracelsus famously said, “All things are poison and nothing is without poison. Solely the dose determines that a thing is not a poison.” (1). Applied to medicines, the statement offers both a warning and a beacon of hope: even the most beneficial substances can be toxic if given at too high a dose, but at the same time, reducing the harmful effects of a drug may require nothing more than lowering the dose. The problem with this philosophy emerges when the harmful and beneficial effects of a drug are inextricably linked, so that reducing the dose to decrease toxicity also diminishes drug efficacy. In the current issue of the *JCI*, Duchatel, Jackson, and colleagues (2) present an elegant solution to this problem: instead of dropping the dose of the effective-but-toxic drug, they combine it with an agent that counteracts the toxicity. The approach resulted in a remarkably effective therapy for an extremely lethal brain tumor: diffuse intrinsic pontine glioma (DIPG).

DIPG is a devastating disease that predominantly affects children and has a median survival of less than one year. So far, most monotherapies have failed to prolong survival of DIPG patients (3). One exception is the mitochondrial ClpP protease activator ONC201, which has resulted in a marked increase in survival in a subset of DIPG patients (4). However, even ONC201 treatment culminates in resistance in most patients (5). To address the challenge of resistance to monotherapies, there have been multiple efforts to use drug combinations, such as ONC201 and the phosphatidylinositol 3-kinase (PI3K) inhibitor paxalisib, or the histone deacetylase inhibitor panobinostat and the proteasome inhibitor marizomib (5, 6)). Unfortunately, combinatorial strategies are often accompanied by severe, dose-limiting toxicities. Interestingly, it has been shown that treatment-related secondary effects may correlate with improved responses to anticancer agents (7), even in the context of brain tumors, where hand and foot skin reactions were more commonly observed in patients with high-grade glioma that had enhanced responses to the multikinase inhibitor regorafenib (8). These observations suggest that it may be more valuable to focus on mitigating the side effects rather than reducing the dose and thereby compromising therapeutic efficacy.

## Overcoming toxicity and resistance

**Figure 1:**
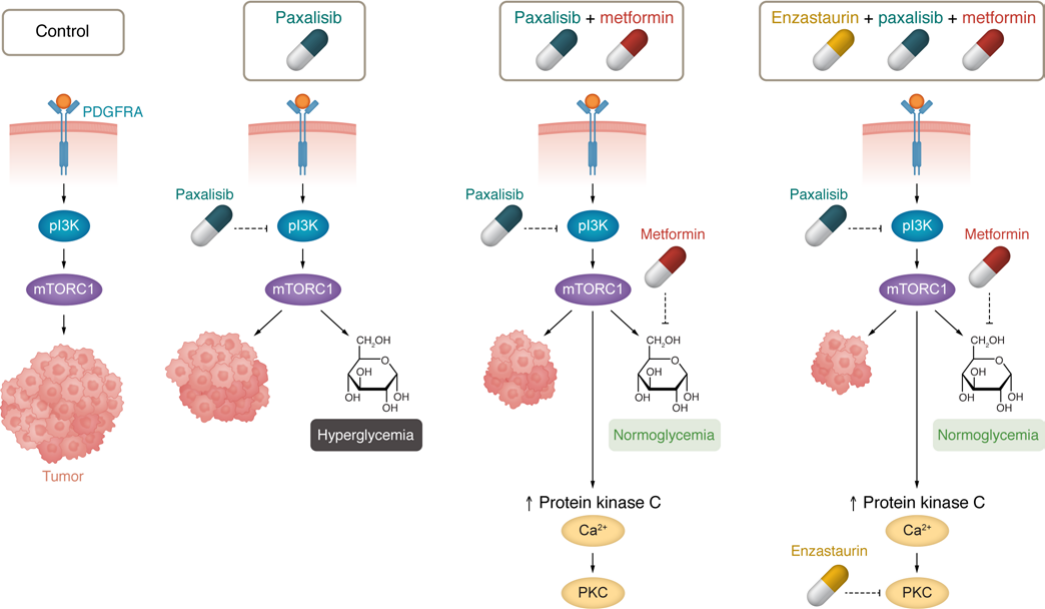
Combinatorial treatment with paxalisib, metformin, and enzastaurin prolongs survival in models of DIPG. Signaling through PDGFRA activates the PI3K/mTOR axis, which promotes tumor growth. Paxalisib effectively targets PI3K, a therapeutic vulnerability in DIPG cells; however, it induces hyperglycemia, which is mitigated by combining it with metformin. DIPG cells escape this dual therapy by upregulation of PKC signaling, which is counteracted by adding enzastaurin as a triple combinatorial strategy. Adapted from a graphic created in BioRender.com (2024).

The treatment regimen developed by Duchatel, Jackson, and colleagues exemplifies this approach. Their strategy included targeting the PI3K/mTOR pathway with paxalisib, mitigating paxalisib-associated toxicity (particularly hyperglycemia) with the antidiabetic drug metformin, and tackling one potential resistance mechanism, namely upregulation of PKC signaling, with the PKC inhibitor enzastaurin (Figure 1) (2).

The authors began by demonstrating the critical importance of the PI3K/mammalian target of rapamycin (mTOR) pathway in DIPG. The role of this pathway has been suggested based on recurrent mutations in PI3K genes and based on frequent activation of PDGF receptors, whose downstream effects are mediated in part by PI3K. The authors confirmed the PI3K/mTOR signaling pathway as a therapeutic vulnerability in various DIPG-derived models by performing CRISPR-Cas9 screens and high-throughput drug screens and pinpointed paxalisib as a potential drug for DIPG irrespective of PI3K mutational status. This last finding is especially interesting, since it highlights a PI3K-pathway dependency across a broad range of patients with DIPG.

To define an optimal dose for paxalisib, Duchatel and Jackson et al. performed pharmacokinetic and pharmacodynamic studies and observed that treating mice twice daily with 5 mg/kg — a dose used in a previous study by the same group (5) — was relatively well-tolerated and had good brainstem penetration, which is critical for efficacy against DIPG. However, Duchatel and Jackson et al. (2) observed that the upregulation of insulin receptor signaling resulted in hyperglycemia and associated symptoms (9). To overcome this problem, they coadministered metformin, an antidiabetic drug with a known and favorable safety profile and showed that it counteracted the activation of the insulin pathway and thereby increased the survival benefit for mice treated with 5 mg/kg paxalisib twice daily (2).

Notably, however, even mice treated with the combination therapy succumbed to the disease, suggesting that this therapy evokes resistance. To gain insight into the mechanisms of resistance, the authors performed phosphoproteomic analysis of treatment-resistant DIPG cells and showed upregulation of the PKC pathway. To counteract this response, they combined paxalisib plus metformin with PKC inhibitors. Specifically, they used enzastaurin, a brain-penetrant, FDA-approved PKC inhibitor that has already been tested in patients with DIPG as a monotherapy. Importantly, they showed additive effects of the combinatorial treatment in vitro and in vivo. There was a survival benefit from the triple therapy (paxalisib-metformin-enzastaurin) compared with either monotherapy with enzastaurin or paxalisib plus metformin (Figure 1); remarkably, the combination of triple therapy with radiation was a safe strategy and prolonged survival in an immunocompetent DIPG model. To understand the mechanism underlying the effects of their therapy, the authors performed spatial transcriptomic and ATAC-Seq analysis of a treated patient-derived xenograft model. This study showed that triple treatment resulted in downregulation of key myelination-associated genes. The authors speculated that this combination strategy targeted a key cell population of these tumors, since DIPG arises from cells of the oligodendroglial lineage that specialize in making myelin (2, 10). Moreover, they observed upregulation of the PDGFRA, JAK-STAT, and TGF-β signaling pathways, identifying additional potential mechanisms of resistance to their treatment regimen (2). These findings pave the way for further improvements in DIPG therapy.

## Clinical implications and future considerations

The use of metformin to reduce the toxicity of PI3K inhibitors is an exciting therapeutic strategy. However, the authors did not fully exclude the possibility that, in their studies, metformin had exerted a direct anticancer effect as well. Based on its effects on mitochondrial function —inhibition of complex 1 and reduction of proton-driven ATP synthesis (11) — and its inhibition of insulin-like growth factor signaling, metformin has been studied as a cytostatic agent in a variety of cancers, including brain tumors (specifically IDH-mutated gliomas) (12). Of note, the doses used by the authors to mitigate the side effects of paxalisib were rather low, and were similar to doses used in the clinic for treatment of diabetes — 175 mg/kg in mice, corresponding to a human dose of 850mg for a 60kg adult (13). In contrast, several trials analyzing the anticancer potential of metformin have used doses of up to 3 g/day (14), more than three times the antidiabetic dose. Noch et al. recently reported on hyperglycemia as a resistance mechanism to paxalisib in glioblastoma, and to overcome this they used metformin at a dose of 200 mg/kg (15), slightly higher than the dose used in Duchatel and Jackson et al. In addition, Noch and colleagues also fed mice a ketogenic diet, an intervention known to reduce hepatic glycogen stores, potentially limiting the release of glucose following PI3K inhibition (15, 16). The resulting Phase II clinical trial for patients with newly diagnosed glioblastoma combines paxalisib with a ketogenic diet and metformin (15). It would be interesting to see if a similar approach is effective for DIPG.

Duchatel, Jackson, and authors pinpoint PDGFRA upregulation as a potential mechanism of resistance to their triple combination therapy (2). PDGFRA is often amplified in DIPG and its expression is particularly prominent in oligodendrocyte precursor–like (OPC-like) cells, a key tumor-propagating cell compartment (10). Recent encouraging studies have identified avapritinib as a potent, brain penetrant PDGFRA inhibitor that is well tolerated and shows good responses in DIPG patients (17). Notably, avapritinib is used as a fourth-line therapy for patients with gastrointestinal tumors that have already developed resistance to tyrosine kinase inhibitors (18), underscoring its potency in targeting PDGRA-driven tumors. Combining this approach with PI3K inhibition, antagonism of the insulin pathway to reduce side effects, and PKC inhibitors could represent an exciting multimodal therapy that targets several key oncogenic pathways in DIPG. In this context, though, it will be essential to perform thorough in vivo experiments addressing the toxicity of such a combination, since even drugs that are well tolerated as monotherapies may induce toxicity when administered in combination.

Treatment of DIPG has hardly changed over the last century. Clinical trials with agents that interfere with key oncogenic signaling pathways have failed to prolong survival for these patients. It is important to understand the plasticity of cancer cells as a key mechanism of resistance and to develop novel combinatorial therapies based on well-designed preclinical studies. Moreover, it is critical to manage drug-related toxicity, not only because it affects patients’ quality of life, but also because it necessitates dose reductions that limit drug efficacy. Jumping into a clinical trial without considering these issues has led to several agents dropping out of the race to tackle DIPG, even though they might have been highly beneficial for patients had they been combined with other agents or had their side effects been mitigated. Duchatel, Jackson, and colleagues introduce a treatment paradigm that should be pursued in both pediatric and adult neurooncology. By carefully studying mechanisms of toxicity and resistance, they developed a treatment strategy that antagonizes hallmark oncogenic pathways, mitigates side effects, and counteracts mechanisms of resistance. We can only hope that these studies will inspire further preclinical research that will ultimately lead to improved outcomes for patients with DIPG and other brain tumors.

## Version 1 03/15/2024

Electronic publication
